# Insights on virulence from the complete genome of *Staphylococcus capitis*

**DOI:** 10.3389/fmicb.2015.00980

**Published:** 2015-09-23

**Authors:** David R. Cameron, Jhih-Hang Jiang, Karl A. Hassan, Liam D. H. Elbourne, Kellie L. Tuck, Ian T. Paulsen, Anton Y. Peleg

**Affiliations:** ^1^Department of Microbiology, Monash UniversityMelbourne, VIC, Australia; ^2^Department of Chemistry and Biomolecular Sciences, Macquarie UniversitySydney, NSW, Australia; ^3^School of Chemistry, Monash UniversityMelbourne, VIC, Australia; ^4^Department of Infectious Diseases, Alfred HospitalMelbourne, VIC, Australia

**Keywords:** coagulase-negative staphylococci, CoNS, SMRT sequencing, genomics, methylation

## Abstract

*Staphylococcus capitis* is an opportunistic pathogen of the coagulase negative staphylococci (CoNS). Functional genomic studies of *S. capitis* have thus far been limited by a lack of available complete genome sequences. Here, we determined the closed *S. capitis* genome and methylome using Single Molecule Real Time (SMRT) sequencing. The strain, AYP1020, harbors a single circular chromosome of 2.44 Mb encoding 2304 predicted proteins, which is the smallest of all complete staphylococcal genomes sequenced to date. AYP1020 harbors two large mobile genetic elements; a plasmid designated pAYP1020 (59.6 Kb) and a prophage, ΦAYP1020 (48.5 Kb). Methylome analysis identified significant adenine methylation across the genome involving two distinct methylation motifs (1972 putative 6-methyladenine (m6A) residues identified). Putative adenine methyltransferases were also identified. Comparative analysis of AYP1020 and the closely related CoNS, *S. epidermidis* RP62a, revealed a host of virulence factors that likely contribute to *S. capitis* pathogenicity, most notably genes important for biofilm formation and a suite of phenol soluble modulins (PSMs); the expression/production of these factors were corroborated by functional assays. The complete *S. capitis* genome will aid future studies on the evolution and pathogenesis of the coagulase negative staphylococci.

## Introduction

*Staphylococcus capitis* was first isolated from human skin in 1975 and classified as a species of the coagulase negative staphylococci (CoNS) (Kloos and Schleifer, 1975). *S. capitis* can be further divided into two subspecies; *capitis* and *ureolyticus* based on urease production and maltose fermentation for the latter (Bannerman and Kloos, 1991). Traditionally considered commensals, many CoNS species are now recognized as opportunistic human pathogens. In fact, multiple studies have found CoNS to be the most frequently isolated organisms from bloodstream infections in intensive care units (N.N.I.S, 1999; Wisplinghoff et al., 2004). *S. capitis* is particularly problematic in neonatal intensive care units causing up to 20% of cases of neonatal sepsis (Van Der Zwet et al., 2002; Rasigade et al., 2012). *S. capitis* is also occasionally associated with native and prosthetic valve endocarditis, as well as hospital-acquired meningitis (Bandres and Darouiche, 1992). Despite the role of *S. capitis* in these infections, very little is known about its pathogenicity. In addition, the treatment of infections caused by *S. capitis* is complicated by the emergence of strains with reduced susceptibility to last line antistaphylococcal agents including vancomycin and linezolid (Cai et al., 2012; Rasigade et al., 2012).

*Staphylococcus epidermidis* is the most frequently studied CoNS species. *S. epidermidis* relies heavily on its ability to form robust biofilms for infection and as such, it typically causes infections associated with indwelling medical devices (Von Eiff et al., 2002; Otto, 2009). *S. epidermidis* produces a range of surface proteins that are important for initial attachment and establishment of biofilm, as well as polysaccharide intercellular adhesin (PIA), which mediates cell to cell adhesion and biofilm accumulation (Mack et al., 1996; Heilmann et al., 1997, 2003). *S. epidermidis* also produces extracellular peptides termed phenol soluble modulins (PSMs), which are proinflammatory and also contribute to biofilm production (Wang et al., 2011; Peschel and Otto, 2013). Whilst biofilm production also appears to be a virulence determinant for *S. capitis* (de Silva et al., 2002), the contributing molecular determinants are less well defined for this species.

Whole genome sequencing (WGS) has become a vital tool in understanding the virulence of pathogenic bacteria. WGS has been used to predict the virulence of *S. epidermidis* RP62a (Gill et al., 2005) and *S. epidermidis* ATCC 12228 (Zhang et al., 2003) by comparing their genomes to that of the most pathogenic staphylococcal species, *S. aureus*. These studies gave powerful insights into genes that likely contribute to *S. epidermidis* virulence, particularly those involved in biofilm formation and immune defense (Zhang et al., 2003; Gill et al., 2005). Subsequently, similar comparative genomics projects have been undertaken for other CoNS species such as *S. saprophyticus* (Kuroda et al., 2005), *S. haemolyticus* (Takeuchi et al., 2005), and *S. lugdunensis* (Heilbronner et al., 2011).

To date, few genome sequences of *S. capitis* are publically available limiting our capacity to predict genes that may be important for *S. capitis* virulence. In the present study, we used Single Molecule Real Time (SMRT) sequencing technology to generate the complete *S. capitis* genome using the clinical bloodstream isolate, AYP1020. SMRT sequencing has emerged as a highly attractive sequencing platform due mainly to its ability to produce long read lengths facilitating efficient and accurate *de novo* complete genome assembly (Chin et al., 2013; Roberts et al., 2013). SMRT sequencing has the added advantage of allowing for the analysis of DNA methylation (Fang et al., 2012). In prokaryotes, DNA methylation has been shown to be important for critical processes including DNA replication and mismatch repair, as well as controlling gene expression and virulence (Heithoff et al., 1999; Marinus and Casadesus, 2009; Katayama et al., 2010). By comparing the *S. capitis* genome with that of *S. epidermidis* RP62a, we have identified factors that likely contribute to *S. capitis* virulence, including genes important for the establishment and maturation of biofilms and a number of putative PSMs. These genetic attributes were then functionally assessed, which showed that *S. capitis* AYP1020 produced biofilms on polymeric surfaces and secreted predicted PSMs in culture supernatants.

## Methods

### Bacterial strains and antibiotic susceptibility testing

*S. capitis* isolate AYP1020 was originally isolated from blood and confirmed to be *S. capitis* by Matrix-Assisted Laser Desorption/Ionization Time of Flight Mass Spectrometry (MALDI-TOF MS) and confirmed to be subsp. *capitis* based on 16S rRNA sequence. After isolation, AYP1020 was cultured on Horse Blood Agar, grown in Heart Infusion Broth (Oxoid) then stored at −80°C in 25% (v/v) Glycerol. *S. epidermidis* RP62a is a biofilm-producing, methicillin-resistant *S. epidermidis* (MRSE) strain isolated from a patient with intravascular catheter-associated sepsis (Christensen et al., 1985). It has a fully sequenced genome (accession number NC_002976) (Gill et al., 2005) and was used in this study for comparative genomic analysis. Antibiotic susceptibility testing was performed using broth dilution for penicillin G, gentamicin, methicillin, erythromycin, and streptomycin according to Clinical Laboratory Standards Institute guidelines (C.L.S.I, 2013).

### SMRT sequencing, assembly and annotation

*S. capitis* AYP1020 genomic DNA was prepared using the QIAGEN Blood and Tissue Kit as per manufacturer's instructions following an initial lysis step in TE buffer (pH 8.0) supplemented with 0.4 mg/ml lysostaphin (Sigma-Aldrich). DNA sequencing was performed using the PacBio RS platform (Pacific Biosciences). A 20-kb library was sequenced using P4-C2 chemistry on 2 SMRT cells yielding 565 Mbp from 92,793 reads. The average read length was 6088 bp with a sequencing depth of 164X. The continuous long reads (CLR) were assembled *de novo* using the PacBio hierarchical genome assembly process (HGAP) and polished using Quiver as described previously (Chin et al., 2013). DNA methylation was determined using the RS_Modification_and_Motif_Analysis protocol within the SMRT Portal v1.3.3. All motifs considered to be modified had a mean modification quality value (QV) > 30 and a mean coverage of >75X. Genome annotation was performed using Prokka (Seemann, 2014). The annotated genome was then manually curated and visualized using Geneious (Biomatters) and Artemis (Rutherford et al., 2000). Prophage were predicted using Phast (Zhou et al., 2011), insertion elements were predicted using ISfinder (Siguier et al., 2006) and putative genomic islands were predicted using Island Viewer (Langille and Brinkman, 2009). The complete genome sequence of *S. capitis* AYP1020 and its plasmid, pAYP1020 were submitted to GenBank with accession numbers CP007601 and CP007602, respectively.

### Comparative genomics

For interspecies comparative analysis, we identified orthologous genes by running a bidirectional BLASTp search between all annotated protein-coding DNA sequences (CDS) for each species. CDS were considered orthologous when amino acid identity was greater than 35 across 75% of the protein, with an *e*-value limit of 1e-30 (Altschul et al., 1997). To confirm that CDSs predicted to be unique in one strain were not due to differences in annotation or due to the presence of paralogous sequences we ran tBLASTn searches of putative unique gene sequences against the chromosomes. Those genes that hit the chromosome with an *e*-value less than 1e-30 were considered non-unique. The degree of BLAST identity and the distribution of Clusters of Orthologous Groups (COGs) were visualized as a circular genome representation using CGview (Tatusov et al., 2000; Grant et al., 2012). Phylogenetic analysis was performed using the multilocus sequence typing (MLST) scheme developed for *S. epidermidis* (Thomas et al., 2007). To compare PSMs, amino acid sequences from *S. capitis* AYP1020 and *S. epidermidis* RP62a were clustered using the unweighted pair group method with arithmetic mean and a phylogenetic tree was generated using Geneious (Biomatters). IcaRADBC amino acid sequences were compared using ClustalW (Larkin et al., 2007).

### Biofilm analysis

Biofilm formation was assessed using scanning electron microscopy (SEM). AYP1020 was grown overnight in tryptic soya broth (TSB, Oxoid) then diluted 1:100 in TSB with 0.25% glucose in a 24 well microtitre plate containing either a polyurethane or silicon coupon (Biosurface Technologies). Biofilms were allowed to mature for 24 h at 37°C with gentle shaking (75 rpm) then coupons were rinsed three times with phosphate buffered saline (PBS). Samples were fixed with 2.5% glutaraldehyde/1M cacodylate buffer followed by 1% osmium tetroxide, then dehydrated with ethanol and hexamethyldisilazane. Biofilms were coated with gold using a Balzers SCD005 sputter coater then visualized using a Hitachi S570 SEM. Biofilms generated by *S. capitis* AYP1020 and *S. epidermidis* RP62a were quantified using 96-well polystyrene microtitre plates (Falcon). Briefly, bacteria were grown as described above, planktonic cells were removed and biofilms were washed three times with PBS. Adherent material was stained using 0.1% crystal violet for 15 min. Crystal violet was emulsified using 96% ethanol and optical densities (570 nm) were determined using an infinite M200 plate reader (Tecan). Biofilms were quantified from four independent experiments and significance was determined by Mann-Whitney *U*-test with a significance level of *P* < 0.05.

### Detection of PSM peptides using high-performance liquid chromatography (HPLC) and mass spectrometry (MS)

PSM peptides released by *S. capitis* strain AYP1020 were detected using reverse-phase HPLC and MS as described previously (Gao et al., 2013). Briefly, filtered culture supernatants were analyzed using an Agilent 1200 series HPLC equipped with a reversed-phase analytical column (Agilent Eclipse XDB-C18 4.6 × 150 mm) (Gao et al., 2013). PSM peptides were eluted with a water/acetonitrile (0.1% trifluoroacetic acid) gradient from 0 to 100% acetonitrile over 28 min at 1 mL per min and detected at 214 nm. The identity of PSMs within isolated fractions was determined based on *m*/*z* ratios compared to the molecular mass of each predicted PSM using a Waters Micromass ZQ mass spectrometer.

## Results and discussion

### Genome summary and general features

**Figure 1:**
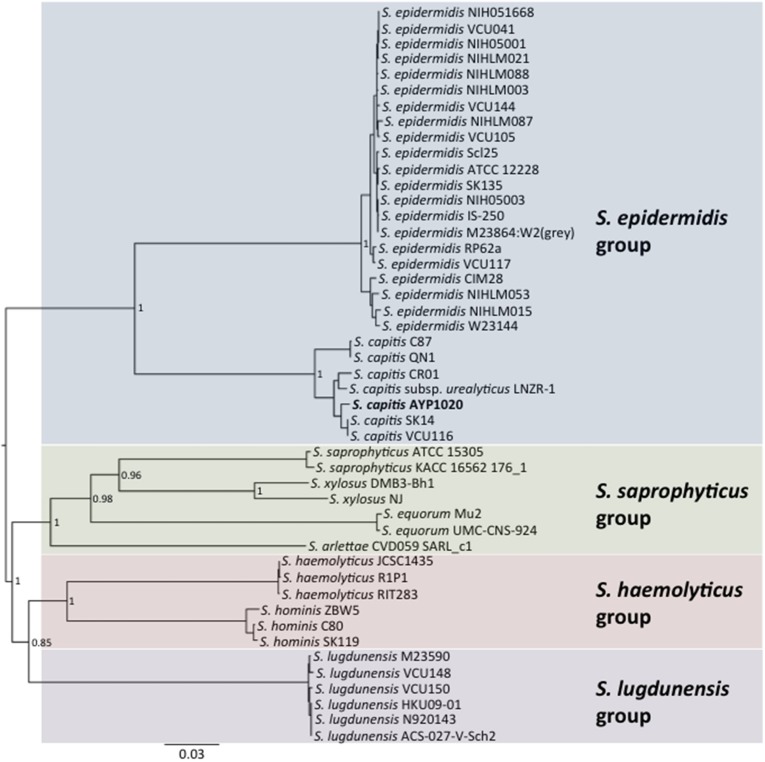
**Neighbor-joining tree showing the relationship between *S. capitis* AYP1020 and other sequenced CoNS strains**. Phylogeny was inferred using the MLST scheme developed for *S. epidermidis*. *S. capitis* AYP1020 is represented in bold.

**Figure 2:**
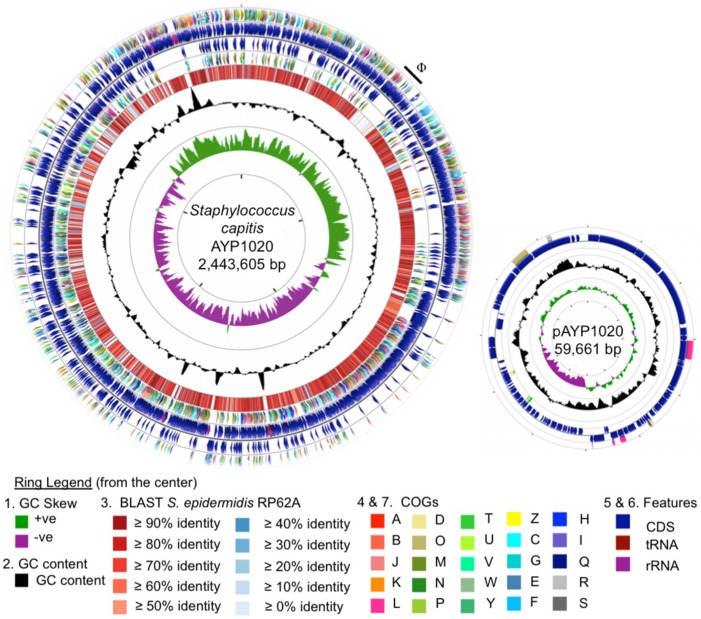
**Circular representation of the *S. capitis* AYP1020 chromosome and its plasmid, pAYP1020**. For the chromosome, the first (innermost) ring indicates the GC skew, followed by the GC content (second ring). The third ring indicates the degree of amino acid identity across all CDS of *S. capitis* AYP1020 compared to *S. epidermidis* RP62a, as determined by BLASTp and scaled according to percent identity as indicated in the key (Altschul et al., 1997). Colored arrows on the fourth and seventh rings represent the following COG categories (Tatusov et al., 2000); A, B, J, K, L, information and storage and processing; D, O, M, N, P, T, U, V, W, Y, Z, cellular processes and signaling; C, G, E, F, H, I, Q, metabolism; R and S, poorly characterized, on the reverse and forward strands, respectively. The fifth and sixth rings represent the CDS (blue), tRNA (maroon), and rRNA (purple) on the reverse and forward strands, respectively. For the genome, the scale displayed in the center of the graphic represents 500 kb. For the plasmid, the scale represents 5 kb. The images were generated using CGview (Grant et al., 2012).

Phylogenetic analysis revealed a close relationship between AYP1020 and other *S. capitis* strains for which genome sequence information was available, particularly *S. capitis* SK14 and *S. capitis* VCU116 (Figure 1). The analysis also shows that *S. capitis* has closer evolutionary links to *S. epidermidis* than other clinically relevant coagulase negative staphylococci (Figure 1). *S. capitis* strain AYP1020 has a 2,443,605 bp chromosome with a GC content of 33.0% (Figure 2). The chromosome is predicted to contain 2304 protein-coding DNA sequences, six rRNA operons, 63 tRNA genes and a single tmRNA (Table 1). *S. capitis* AYP1020 represents the smallest genome of all closed staphylococcal genomes sequenced to date, with the fewest predicted CDS.

**Table 1:** **General genomic features of *S. capitis* strain AYP1020 compared with *S. epidermidis* RP62a**.

**Feature**	***S. capitis* AYP1020**	***S. epidermidis* RP62a^a^**
Size (bp)	2,443,605	2,626,530
Number of CDS	2304	2490
tmRNA	1	1
tRNA	63	61
rRNA 16S	6	6
rRNA 23S	6	6
rRNA 5S	7	7
G + C content	33.0%	32.1%
Plasmid	59,661 bp	27,310 bp
Prophage	1	1
IS elements	1	64^b^

a *Described in Gill et al. (2005)*.

b *Only 18 are predicted to be intact (Rosenstein et al., 2009)*.

*S. capitis* strain AYP1020 has a large plasmid designated pAYP1020. The plasmid is 59.6 Kb in size, has a GC content of 30.3% and is similar to the previously sequenced plasmid, SAP020A (75% coverage, 77% identity) from *Staphylococcus* sp. CDC3 (GenBank: NC_013373.1). The plasmid contains 71 predicted CDS, the majority of which encode proteins with no assigned function (54 CDS) (Figure 2). The AYP1020 chromosome also has a 48.5 Kb prophage designated ΦAYP1020 (Figure 2), encoding 24 typical phage proteins including integrase, terminase, portal, head, tail, and lysins, *attR* and *attL* sites plus an additional 30 hypothetical proteins.

### Base modifications

SMRT technology allows for the genome-wide detection of modified nucleotides based on the rate by which DNA polymerase incorporates bases during sequencing (Roberts et al., 2013). Analysis of polymerase kinetic profiles identified 1972 m6A modifications (distribution shown in Figure S1) that were associated with two predicted type I adenine methyltransferase recognition motifs: 5′-CAAN_6_*T*GG-3′ and 5′-CTAN_7_*T*NYC-3′ (modified bases are underlined and modified bases on the opposite strand are italicized). Adenine modification correlated with the presence of four putative adenine methyltransferases in the AYP1020 genome (AYP1020_0291, AYP1020_0991, AYP1020_1852, and AYP1020_1992). In contrast, no cytosine methylation was detected correlating with the absence of predicted cytosine methyltransferase genes.

The overall distribution of putative methylated sites across the AYP1020 chromosome appeared to be random (Figure S1). However, in line with the predicted involvement of adenine methytransferases, regions of higher GC% contained fewer methylated sites. Aside from regions with higher GC%, methylation did not correlate strongly with predicted regions of horizontally acquired DNA (Figure S1). For example, the ΦAYP1020 prophage was methylated with an apparent equal density to the remainder of the AYP1020 chromosomal sequence. Assuming selective pressure for the accumulation of methylation sites within horizontally acquired DNA over time, this observation may suggest that the rate of sequence amelioration is rapid in AYP1020, or that horizontally acquired DNA was obtained from an organism harboring similar methyltransferases. In contrast to the prophage, the pAYP1020 plasmid sequence contained a low methylation site density (Figure S1), which could indicate that this plasmid is subject to different selective pressures than the host chromosome, or that it was relatively recently acquired within AYP1020.

### Comparative genomic analysis of AYP1020 and *S. epidermidis* RP62a

**Figure 3:**
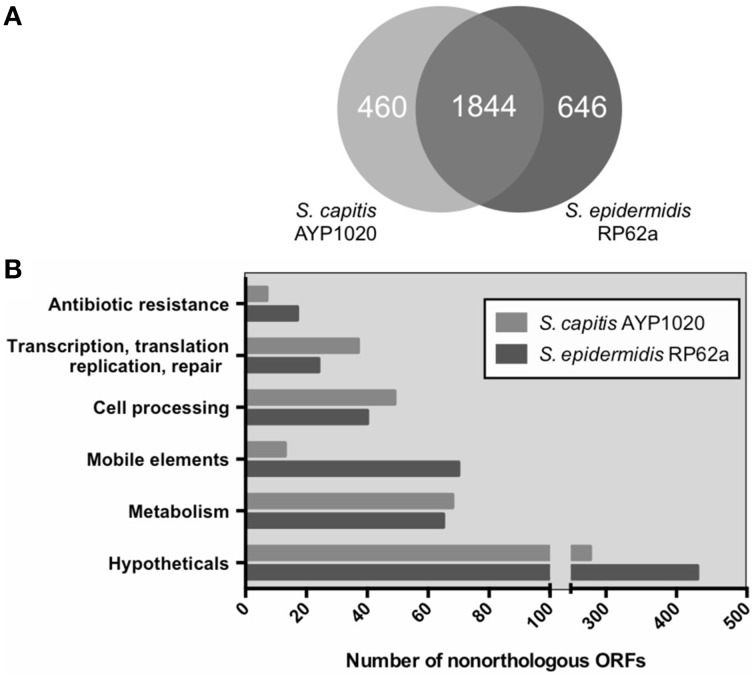
**Orthologous classification of CDS of *S. capitis* AYP1020 compared to *S. epidermidis* RP62a**. Orthologs were defined by bidirectional BLASTp with an *e*-value cut off of 10e-30 **(A)**. Non-orthologous CDS were grouped based on COG functional categories **(B)**.

*S. epidermidis* (RP62a) was chosen for comparative analysis as it is a close relative of *S. capitis* and it is the most clinically important CoNS (Figure 1) (Poyart et al., 2001; Otto, 2009). Using the Artemis Comparison Tool, we found the degree of synteny between *S. capitis* AYP1020 and RP62a to be high, with few significant genomic rearrangements detected (Figure S2). We used BLASTp to compare all CDS sequences of AYP1020 with *S. epidermidis* RP62a and identified a core set of 1844 genes present in both species, which represents 80% of the *S. capitis* genome (Figure 3A). We identified 480 and 646 non-orthologous genes for *S. capitis* and *S. epidermidis*, respectively (Figure 3A). When the non-orthologous genes were categorized based on function, we found that *S. epidermidis* RP62a had an enhanced repertoire of genes related to mobile genetic elements (Figure 3B), which are largely accounted for by IS elements. The genome of AYP1020 encodes only one predicted IS element compared to the genome of *S. epidermidis* RP62a that has 64 predicted IS elements (18 intact), as well as five transposons (Gill et al., 2005; Takeuchi et al., 2005). Importantly, key antibiotic resistance determinants are carried on mobile elements in RP62a and these are absent from AYP1020. These include penicillinase encoded by *blaZ*, as well as SCC*mec*, which not only harbors determinants important for resistance to methicillin, but also aminoglycosides and macrolides (Table 2) (Gill et al., 2005). The absence of these genes in AYP1020 correlates with the highly antibiotic susceptible phenotype of this *S. capitis* isolate (Table 2). Multidrug-resistant strains, however, are emerging, with the majority of clinical strains resistant to penicillin and methicillin, and strains with reduced susceptibility to vancomycin also emerging (Ma et al., 2011; Rasigade et al., 2012). Further sequencing projects of multi-drug resistant isolates is required to determine the genetic factors facilitating antibiotic resistance in *S. capitis*. One such study characterized a novel SCC*mec* element in pulsotype NRCS-A, which harbored genes not only important for methicillin resistance, but also resistance to cadmium, arsenic, and copper (Martins Simões et al., 2013).

**Table 2:** **Comparison of antibiotic resistance profiles of *S. capitis* AYP1020 and *S. epidermidis* RP62a and their associated mobile elements**.

**Antibiotic**	**Resistance gene (s)**			**AYP1020**		**RP62a**	
	**Gene**	**Product**	**Element**	**Presence**	**MIC (μg/mL)**	**Presence**	**MIC (μg/mL)**
Penicillin	*blaZ*	β-Lactamase	Plasmid	–	< 0.25	+	32
Methicillin	*mecA*	Penicillin-binding protein 2′	SCC*mec*	–	2	+	>128
Erythromycin	*ermA*	rRNA adenine *N*-6-methyltransferase	Tn554, SCC*mec*	–	< 0.25	+	>128
Gentamicin	*aacA*	6′-aminoglycoside *N*-acetyltransferase	Phage	–	< 0.25	+	64
Streptomycin	*aadE*,	Aminoglycoside 6-adenylyltransferase	Plasmid	–	2	+	>128
	*aphA*	Aminoglycoside 3′-phosphotransferase	Plasmid	–		+	
	*spc*	Streptomycin 3″-adenylyltransferase	Tn554, SCC*mec*	–		+	

### Insights into *S. capitis* virulence and biofilm production

Importantly, among the CDS orthologs between *S. capitis* and *S. epidermidis*, we identified a number of genes encoding for virulence factors that likely contribute to *S. capitis* pathogenicity (Table 3). CoNS species including *S. capitis* and *S. epidermidis* have a lower virulence potential compared to the most virulent staphylococcal species, *S. aureus* (Massey et al., 2006; Otto, 2009). As such, *S. capitis* and *S. epidermidis* do not code for the extensive suite of exotoxins that would be associated with more invasive and severe infections caused by *S. aureus*. *S. capitis* does however, encode for a number of factors predicted to be important for biofilm production, persistence and immune evasion.

**Table 3:** **Orthologous virulence factors of *S. capitis* strain AYP1020 and *S. epidermidis* strain RP62a**.

**Function**	**Gene (s)**	**Product (s)**	**Locus**	
			***S. capitis* AYP1020**	***S. epidermidis* RP62A**
Global regulators	*agrADCB*	Accessory gene regulator	1223-6	1490-3
	*sarARVZ*	Staphylococcal accessory regulator	2371	0274
			1469	1876
			1442	1849
			1576	1979
	*saeRS*	SaeRS two-component regulatory system	0073–4	0364–5
	*arlRS*	ArlRS two-component regulatory system	0725–6	0988–9
	*rot*	Repressor of toxins	1043	1322
	*sigB*	RNA polymerase sigma factor B	1257	1677
Biofilm/PNAG	*icaRADBC*	Intercellular adhesion proteins	1906–1910	2292–6
PGA	*capDACB*	Capsule biosynthesis proteins	1692	2103
			1694–6	2105–7
Exoenyzmes	*hlb*	Beta Hemolysin	1985	2544
		Hemolysin III	1362	1769
		Hemolysin	–	2258
	*clpP*	Clp protease, proteolytic subunit	0135	0436
	*clpBCX*	Clp protease, ATP binding subunits	0244	0564
			2273	0165
			0957	1238
	*sspA*	Serine V8 protease	–	1397
	*sspB*	Cysteine protease	–	2390
	*sspC*	Cysteine protease	–	2391
	*sepA*	Metalloprotease elastase	1844	2252
		Zinc metalloprotease	0571	0829
		Serine protease, putative	0279	0611
	*htrA*	HtrA like protease, putative	1007	1292
	*splE*	Serine protease	1979	–
		Serine protease	2166	2401
	*lip*	Lipase	1943	2336
	*geh*	Lipase	–	0018
	*geh1*	Lipase	1911	2297
	*geh2*	Lipase	2083	2388
	*lipA*	Lipase/esterase	0022	0309
		Esterase	1534	1941
		Esterase	1701	2109
Pro-inflammatory peptides	*psmα*	Phenol Soluble modulin alpha	2169	0083
	*psmδ*	Phenol Soluble modulin delta	2168	0082
	*psmβ1a*	Phenol Soluble modulin beta 1a	0480	0738/9
	*psmβ1c*	Phenol Soluble modulin beta 1b	0481	–
	*psmβ1d*	Phenol Soluble modulin beta 1c	0482	–
	*psmβ2*	Phenol Soluble modulin beta 2	0479	0737
	*psmβ3*	Phenol Soluble modulin beta 3	–	0736
	*hld*	Delta Hemolysin	1222	1489
Surface proteins/adhesins	*fbe*	fibronectin binding protein A	0515	0775
	*atlE*	Bifunctional autolysin	0374	0636
	*pls*	Plasmin-sensitive protein	2027	–
	*aap*	Accumulation associated protein	–	2398
	*ebh*	Cell wall associated fibronectin binding protein	0751	1011
	*sesA*	Cell wall surface anchor protein	1036–7	1316
	*sesB*	Cell wall surface anchor protein	1776	2162
	*sesC*	Cell wall surface anchor protein	1864	2264
	*sesG*	Cell wall surface anchor protein	1214	1482
	*ebp*	Elastin binding protein	–	1048
	*bhp/bap*	Cell wall associated biofilm protein	–	2392
MSCRAMMs	*sdrX*	SdrX	1833	–
	*sdrZL*	SdrZL	1219	–
	*sdrH*	SdrH protein		1487
	*sdrF*	SdrF protein	–	0026
	*sdrG*	SdrG protein	–	0207

*MSCRAMM, microbial surface component recognizing adhesive molecule; PGA, poly-γ-glutamic acid; PNAG, poly-N-acetlyglucosamine*.

^a^Locus tags AYP1020_

*^b^Locus tages SERP_ (Gill et al., 2005)*.

**Figure 4:**
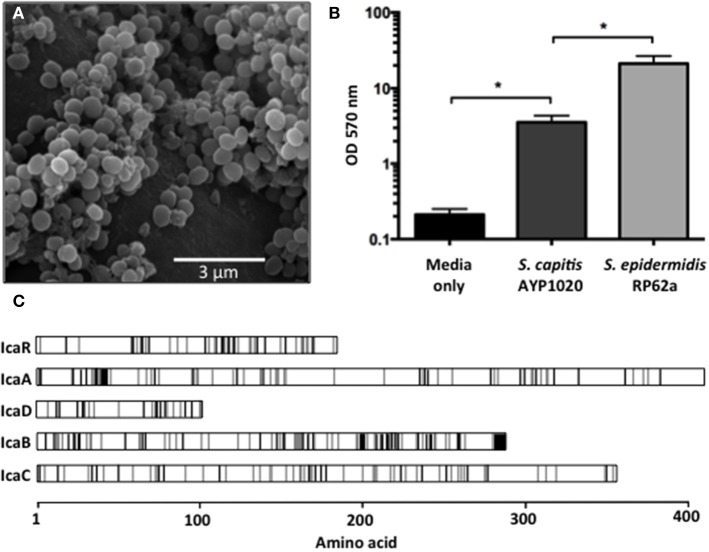
**Biofilm formation of *S. capitis* AYP1020**. Scanning electron micrograph of biofilm formed by *S. capitis* AYP1020 on polyurethane at 10,000X magnification **(A)**. Biofilm was quantified on polystyrene microtitre plates. Data are expressed as mean ± SEM (^*^P < 0.05) **(B)**. Schematic ClustalW alignment revealed high similarity (71–83% identity) between the IcaRADBC proteins of *S. capitis* AYP1020 and those of *S. epidermidis*. White regions represent the same amino acid, gray regions represent similar amino acids and black regions represent non-similar amino acids **(C)**.

Biofilm formation is highly important for CoNS pathogenicity as they allow bacteria to colonize abiotic surfaces such as indwelling medical devices, which helps to establish infections within the host (Von Eiff et al., 2002). Using SEM, we observed *S. capitis* AYP1020 biofilms on multiple polymeric surfaces including polyurethane (Figure 4A) and silicone (data not shown). Whilst *S. capitis* AYP1020 was shown to generate biofilm, the relative production of biofilm was six-fold lower than that of *S. epidermidis* RP62a (Figure 4B). In *S. epidermidis*, the bifunctional autolysin, AtlE has been shown to be important for initial biofilm attachment to polymeric surfaces (Heilmann et al., 1997). Further, fibronectin binding proteins Fbe and Ebh are important for binding to host extracellular matrix proteins, which coat medical devices upon implantation (Nilsson et al., 1998; Von Eiff et al., 2002; Williams et al., 2002). The molecular mechanisms contributing to *S. capitis* surface adhesion are less well defined. A study highlighted the importance of microbial surface component recognizing adhesive molecules (MSCRAMMs) SdrX (Liu et al., 2004) and the “SdrZ like” protein SdrZL, each of which are present in the AYP1020 genome. The homologs of AtlE (70% amino acid identity), Fbe (88% amino acid identity), and Ebh (57% amino acid identity) were also identified. Further studies are required to investigate the role of these proteins in *S. capitis* adhesion.

Proceeding attachment, biofilm accumulation in staphylococci typically occurs via the production of a poly-*N*-acetlyglucosamine (PNAG) homopolymer, which facilitates intercellular adhesion (also referred to as polysaccharide intercellular adhesion, PIA) (Mack et al., 1994). PNAG production is dependent on the *ica* locus, which has been described for *S. capitis*, and is also present in AYP1020 (Heilmann et al., 1996; de Silva et al., 2002). The genetic arrangement and predicted amino acid sequence for the Ica system was similar between *S. capitis* AYP1020 and *S. epidermidis* RP62a (Figure 4C). In addition, a recent study showed that *S. capitis* biofilms could be disrupted by the addition of proteinase K, suggesting that protein is also important for bacterial accumulation in *S. capitis* (Greco-Stewart et al., 2013). The plasmin-sensitive protein Pls of AYP1020 is a likely contributor to the proteinaceous nature of *S. capitis* biofilms as it promotes cell-cell interaction and shares similar domain structure and sequence homology with the accumulation-associated protein (Aap) of *S. epidermidis* (30% amino acid identity) and SasG of *S. aureus* (29% amino acid identity), each of which are important for biofilm accumulation (Hussain et al., 1997; Huesca et al., 2002; Geoghegan et al., 2010).

Along with PNAG, the AYP1020 genome also encodes the *cap* operon, which mediates the production of a second exopolysaccharide, poly-γ-glutamic acid (PGA). In *S. epidermidis*, PGA has an important role in host immune evasion, more specifically, resistance to host antimicrobial peptides and reduced susceptibility to phagocytosis (Kocianova et al., 2005).

### Phenol soluble modulins and exoproteins

**Figure 5:**
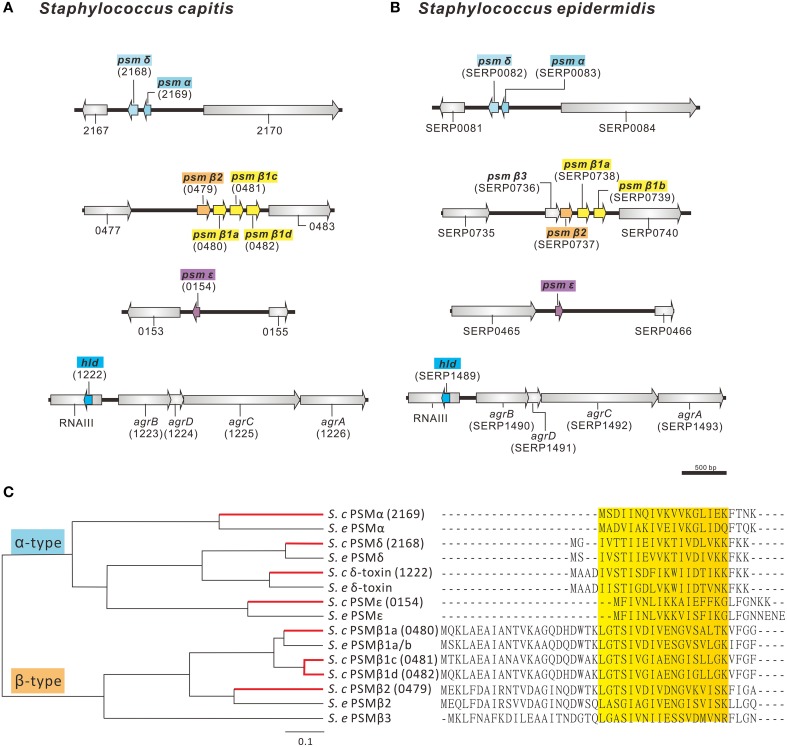
**Predicted phenol-soluble modulin (PSM) genes and amino acid sequences in *S. capitis* AYP1020**. The genetic arrangement of phenol soluble modulin genes in AYP1020 **(A)** is highly similar when compared to *S. epidermidis* RP62a **(B)**. In each case, PSMs are found on 4 distinct genetic loci. Predicted PSMs of *S. capitis* have high amino acid sequence identity when compared to *S. epidermidis* RP62a **(C)**. AYP1020 has both α- and β-type PSMs, which are classed based on length. Each PSM contains an amphipathic α-helix, which is highlighted in yellow. AYP1020 has three distinct PSM β1 genes (a, c, and d), whereas RP62a has two identical copies of PSM β1 (a and b).

PSMs are secreted amphipathic peptides that appear to have multiple functions in the pathogenicity of staphylococci (Wang et al., 2007b; Peschel and Otto, 2013). PSMs have been shown to be pro-inflammatory and possess cytolytic properties, contribute to biofilm development and have anti-microbial activity, as shown by selective *Streptococcus pyogenes* killing (Yao et al., 2005; Wang et al., 2007b; Cogen et al., 2010). The AYP1020 genome encodes for a number of peptides with high sequence similarity to the PSMs of *S. epidermidis* (Figures 5A,B). Like *S. epidermidis, S. capitis* AYP1020 encodes four α-type PSMs and four β-type PSMs (Figure 5C). The β-type PSMs are present in an operon, whereas the α-type PSMs are found in three distinct genomic locations. The *hld* gene, encoding the α-type PSM δ-toxin, is present within RNAIII, the key regulatory molecule of the accessory gene regulator system, Agr (Recsei et al., 1986). PSM peptides have an amphipathic α-helical structure, which causes longer retention times during liquid chromatography compared to most other proteins in culture supernatants (Joo and Otto, 2014).

To determine if the predicted PSM peptides were expressed in AYP1020, we analyzed filtered supernatants using reverse phase-HPLC followed by mass spectrometry. The *m*/*z* ratios of each peptide peak determined by mass spectrometry were correlated to the predicted mass of each predicted PSM peptide (Table 4). The *m*/*z* ratios also indicated that most types of PSMs detected were *N*-terminal formylated excluding PSMβ2 and PSMε. Given the toxic potential of PSM peptides, future research is required to determine the importance of these peptides to *S. capitis* physiology and pathogenesis.

**Table 4:** **Predicted PSMs of *S. capitis* strain AYP1020 were detected in the culture supernatant using HPLC followed by mass spectrometry**.

**PSM**	**Predicted amino acid sequence^a^**	**Molecular Weight^b^**	***m*/*z* of peaks (+ H^+^)^c^**	**Retention time (min)**
α	fMSDIINQIVKVVKGLIEKFTNK	2547.1	1274.2 (+2)	21.3
			849.8 (+3)	
			637.4 (+4)	
β1a	fMQKLAEAIANTVKAGQDHDWTKLGTSIVDIVENGVSALTKVFGG	4643.3	1548.2 (+3)	20.5
			1161.4 (+4)	
			929.7 (+5)	
β1c	fMTKLAEAIANAVKAGQDQDWAKLGTSIVGIAENGISLLGKVFGF	4563.3	1521.9 (+3)	23.5
			1141.6 (+4)	
			913.4 (+5)	
β1d	fMQKLAEAIANTVKAGQDHDWAKLGTSIVGIAENGIGLLGKVFGF	4599.3	1150.3 (+4)	19.5
			920.3 (+5)	
β2	MEKLFDAIRNTVDAGINQDWTKLGTSIVDIVDNGVKVISKFIGA	4822.5	1608.7 (+3)	25.2
			1206.5 (+4)	
δ	MGIVTTIIEIVKTIVDLVKKFKK	2617.3	872.5 (+3)	22.5
			654.6 (+4)	
	fMGIVTTIIEIVKTIVDLVKKFKK	2646.3	1323.7 (+2)	27
			882.9 (+3)	
ε	MFIVNLIKKAIEFFKGLFGNKK	2586.2	862.7 (+3)	17.5
δ-toxin	MAADIVSTISDFIKWIIDTIKKFKK	2912.5	971.6 (+3)	19.5
			729.2 (+4)	
	fMAADIVSTISDFIKWIIDTIKKFKK	2941.5	1471.2 (+2)	25.2
			981.3 (+3)	

a *Formylation of the amino-terminus is indicated as f*.

b *The molecular weight is calculated based on predicted amino acid sequence*.

c *The numbers of protons added to the peptides are suggested*.

The majority of PSMs have been shown to be regulated by Agr (Wang et al., 2007b). Aside from Agr, AYP1020 encodes a number of systems important for the regulation of virulence in staphylococci including the staphylococcal accessory regulator, Sar, the repressor of toxins, Rot and the two component regulatory systems, SaeRS and ArlRS (Table 3) (Cheung et al., 1992; Giraudo et al., 1994; McNamara et al., 2000; Fournier et al., 2001).

*S. capitis* AYP1020 also encodes for a suite of exoproteins that likely contribute to infection. These include proteases such as ClpP, which contributes to biofilm formation and SepA, which has been shown to degrade host antimicrobial peptides in CoNS (Wang et al., 2007a; Cheung et al., 2010), as well as hemolysins, lipases, and esterases. These proteins likely facilitate immune evasion, host colonization and persistence, as opposed to acute disease facilitated by typical exoproteins of the more virulent staphylococcal species, *S. aureus* (Otto, 2009).

In summary, we have generated the closed genome and methylome for *S. capitis* strain AYP1020 using SMRT sequence technology. Our study focused on genes that are predicted to be important for *S. capitis* pathogenicity, based on their role in the closely related opportunistic pathogen, *S. epidermidis*. Having a reliable reference genome invites the sequencing of more diverse *S. capitis* isolates, which would provide further insights into the evolution of virulence and antibiotic resistance in this emerging pathogen.

## Financial support

This work was supported by an Australian Postgraduate Award and a Monash University Postgraduate Publication Award to DC. and an Australian National Health and Medical Research Council (NHMRC) R.D. Wright Career Development Fellowship (APP1047916) and Project Grant (APP1047918) to AP.

### Conflict of interest statement

The authors declare that the research was conducted in the absence of any commercial or financial relationships that could be construed as a potential conflict of interest.
