# Supplementary material for: Insights on virulence from the complete genome of Staphylococcus capitis
Source: Front Microbiol. 2015 Sep 23;6:980. doi: 10.3389/fmicb.2015.00980 (PMC4585213; doi:10.3389/fmicb.2015.00980)
Supplement: Supplementary file 1 [file Presentation1.PPTX]

## Slide 1
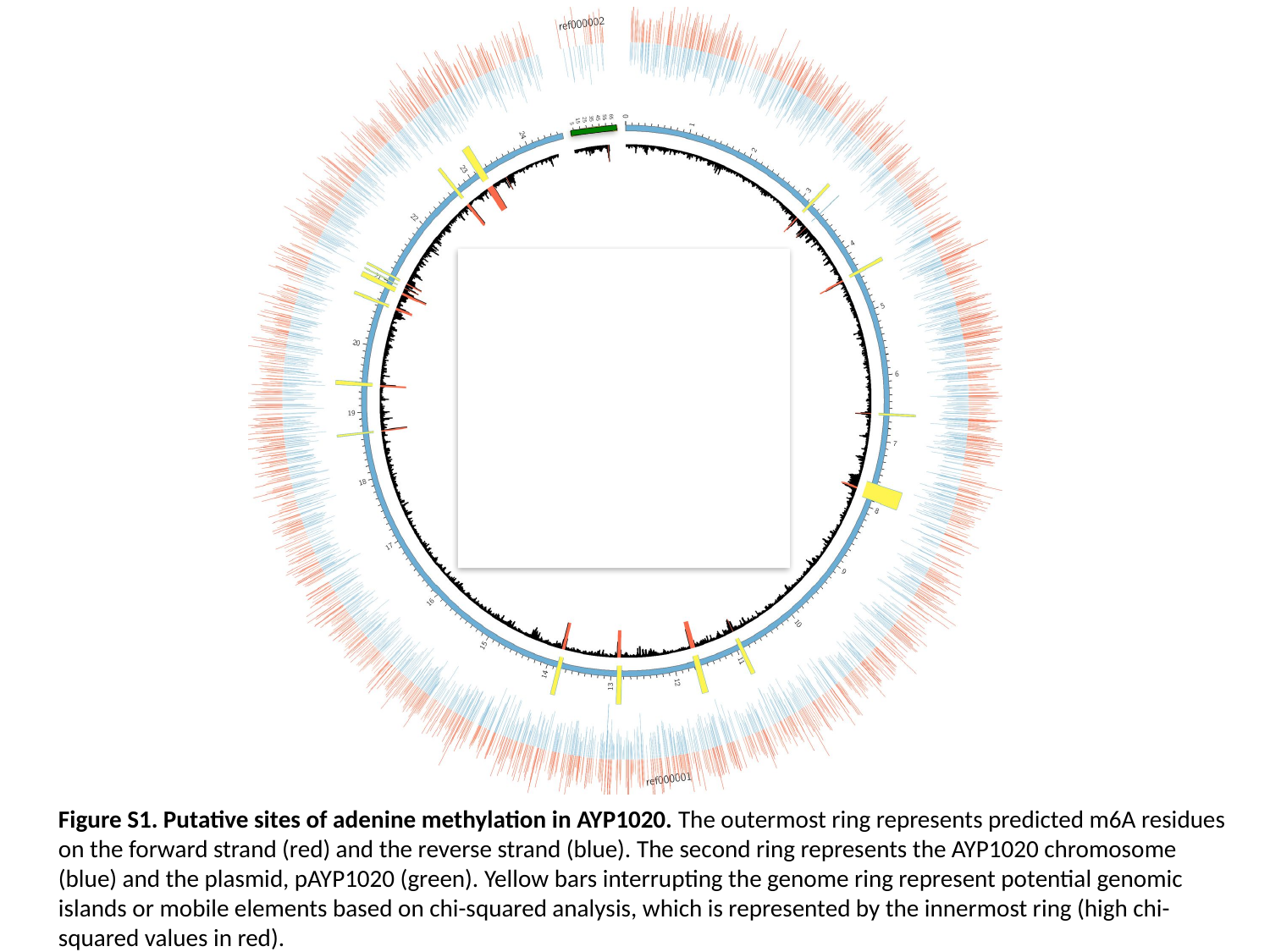

Figure S1. Putative sites of adenine methylation in AYP1020. The outermost ring represents predicted m6A residues on the forward strand (red) and the reverse strand (blue). The second ring represents the AYP1020 chromosome (blue) and the plasmid, pAYP1020 (green). Yellow bars interrupting the genome ring represent potential genomic islands or mobile elements based on chi-squared analysis, which is represented by the innermost ring (high chi-squared values in red).

## Slide 2
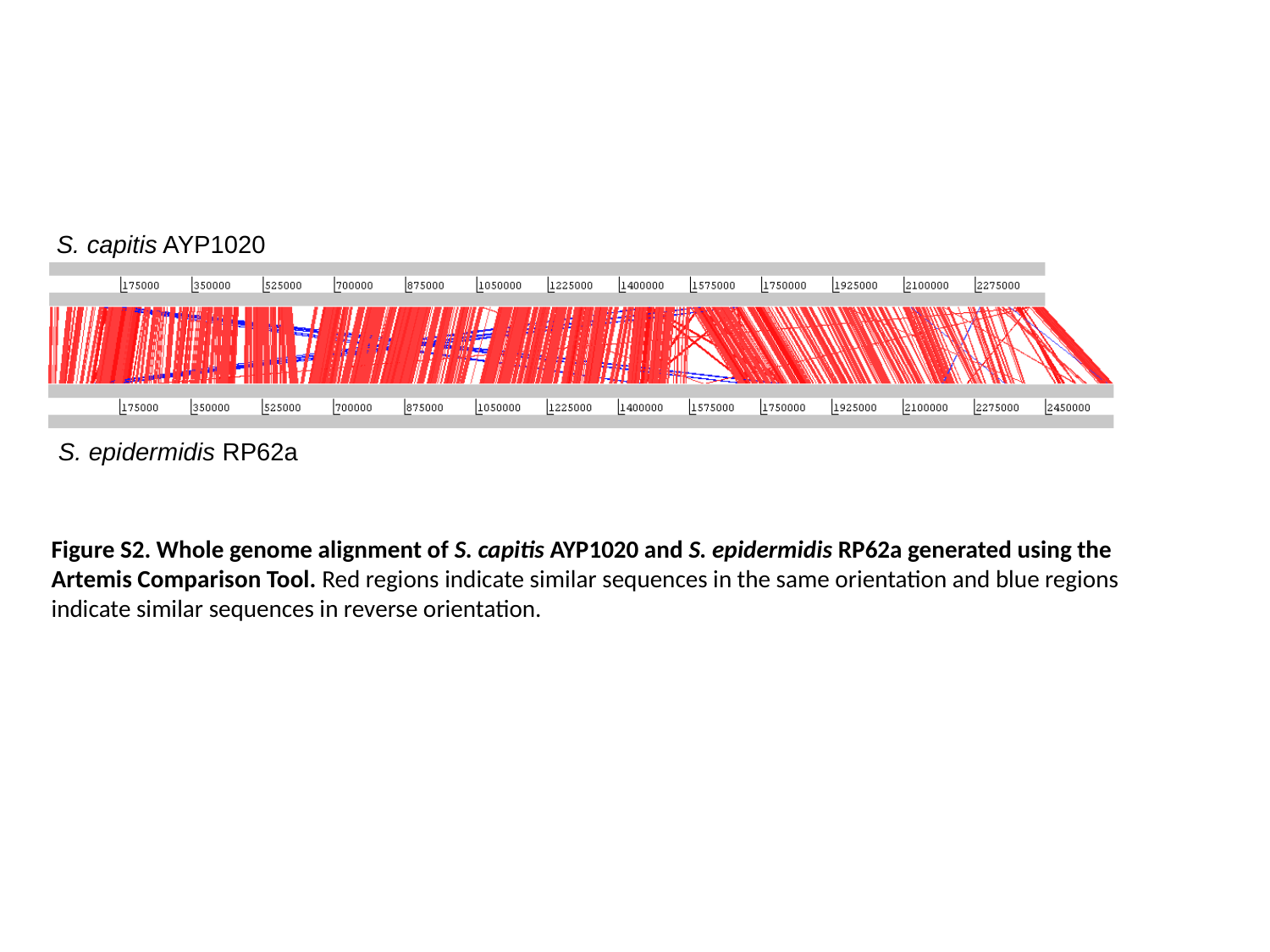

S. capitis AYP1020
S. epidermidis RP62a
Figure S2. Whole genome alignment of S. capitis AYP1020 and S. epidermidis RP62a generated using the Artemis Comparison Tool. Red regions indicate similar sequences in the same orientation and blue regions indicate similar sequences in reverse orientation.
